# Supplementary figures and images for: Expansion of myeloid suppressor cells and suppression of Lassa virus-specific T cells during fatal Lassa fever
Source: PLoS Pathog. 2025 Apr 17;21(4):e1013111. doi: 10.1371/journal.ppat.1013111 (PMC12040235; doi:10.1371/journal.ppat.1013111)

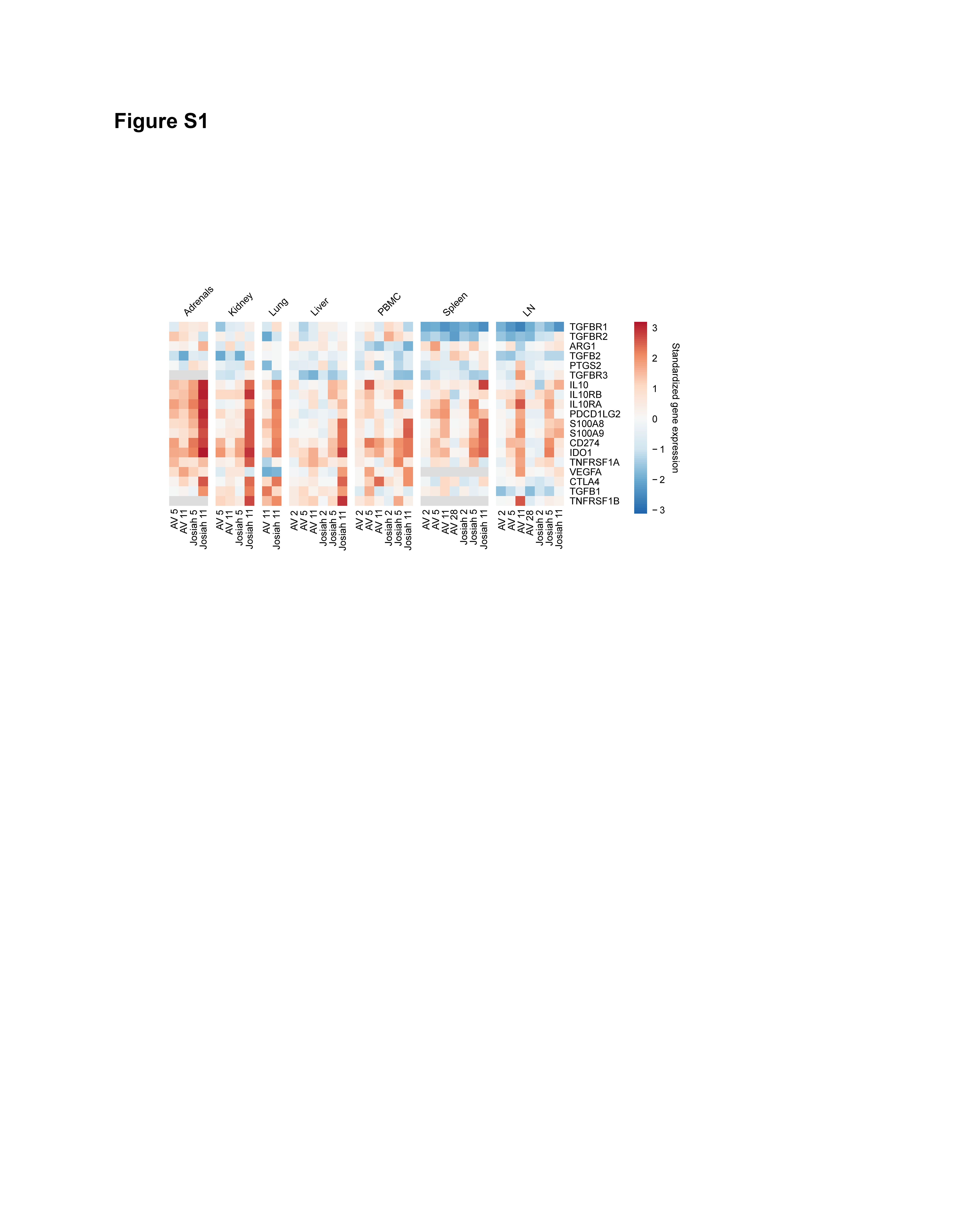

Supplement: S1 Fig — Individual gene expression is averaged in each group, normalized by the non-infected condition, and color-coded according to the scale present on the right-hand side (n = 3 for each condition). (TIF) [file ppat.1013111.s001.tif]

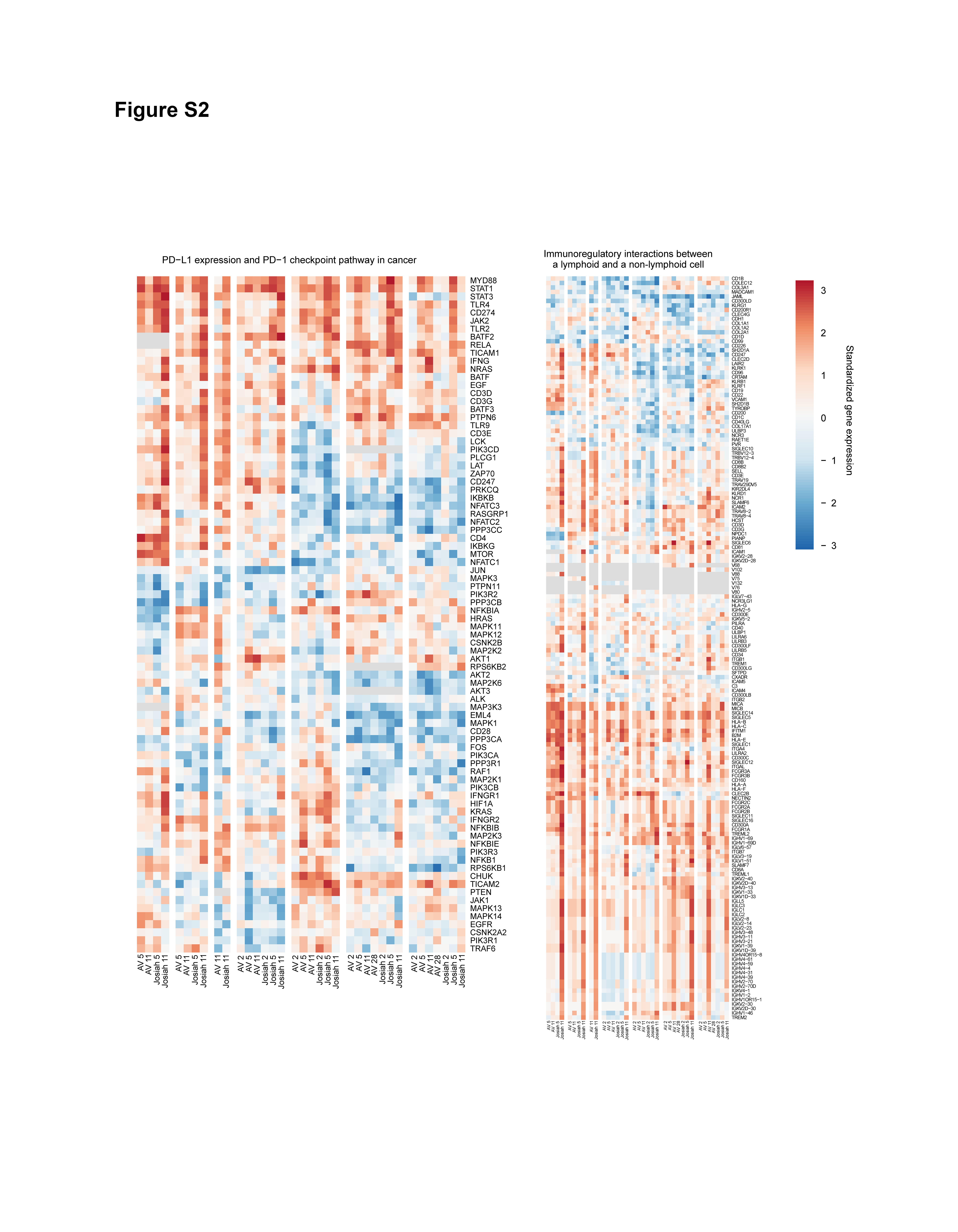

Supplement: S2 Fig — Individual gene expression is averaged in each group, normalized by the non-infected condition, and color-coded according to the scale present on the right-hand side (n = 3 for each condition). (TIF) [file ppat.1013111.s002.tif]

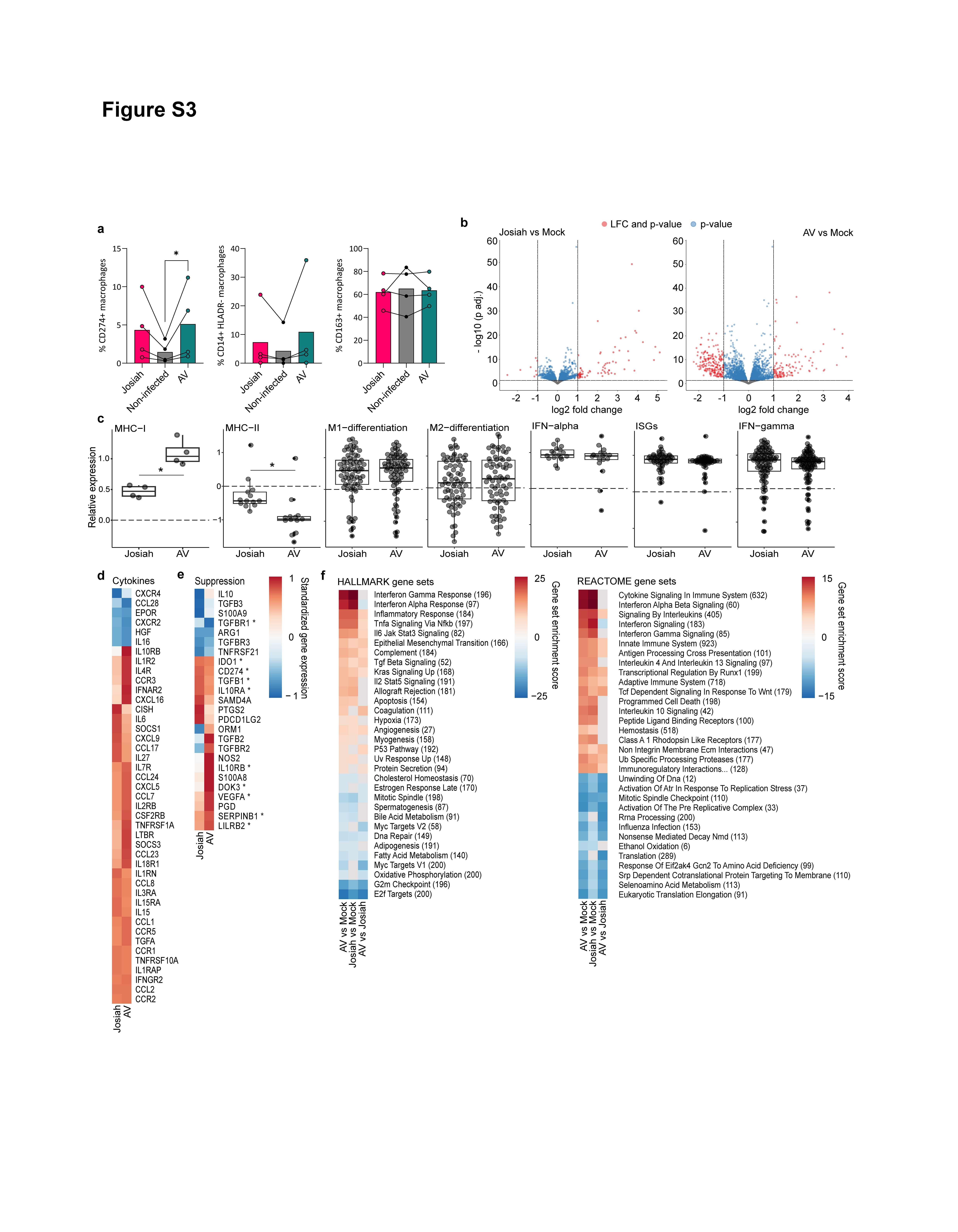

Supplement: S3 Fig — Comparison of the percentage of CD274+, CD14+ HLA-DR-, and CD163+ macrophages in infected and non-infected conditions. A black line connects values obtained from the same human donor and the mean of each condition is presented. According to the result of a D’Agostino-Pearson normality test, we performed a paired one-way ANOVA with Tukey’s test or a non-parametric Friedman’s test with Dunn’s test for multiple comparisons. B. Volcano plot showing the transcriptomic modifications induced by AV or Josiah infection in macrophages. Each gene is represented by a point, blue for an adjusted p-value < 0.05, red for an adjusted p-value < 0.05 and a | log fold change| > 1, or gray when none of the conditions are met. C. Boxplots of the expression of several gene sets relative to the non-infected value. Data presentation and statistical analysis are as in S1 Fig, adding the representation of individual genes in gray circles. D-E. Heatmaps of the expression of gene sets “Cytokines” and “Immunosuppression”. Data are averaged according to the viral strain, normalized by the non-infected value, and color-coded. For the “Cytokines” gene set, all genes shown are significantly differentially expressed (sDE) between at least one of the viral strains and the non-infected condition are presented, whereas all genes of the “Immunosuppression” gene set are presented and sDE genes are labeled by an asterisk. F. Heatmaps of the gene set enrichment score of canonical pathways from the HALLMARK and REACTOME gene sets collections. Only pathways that are significantly different for at least two comparisons are shown. For A to F, data represent four human donors. (TIF) [file ppat.1013111.s003.tif]

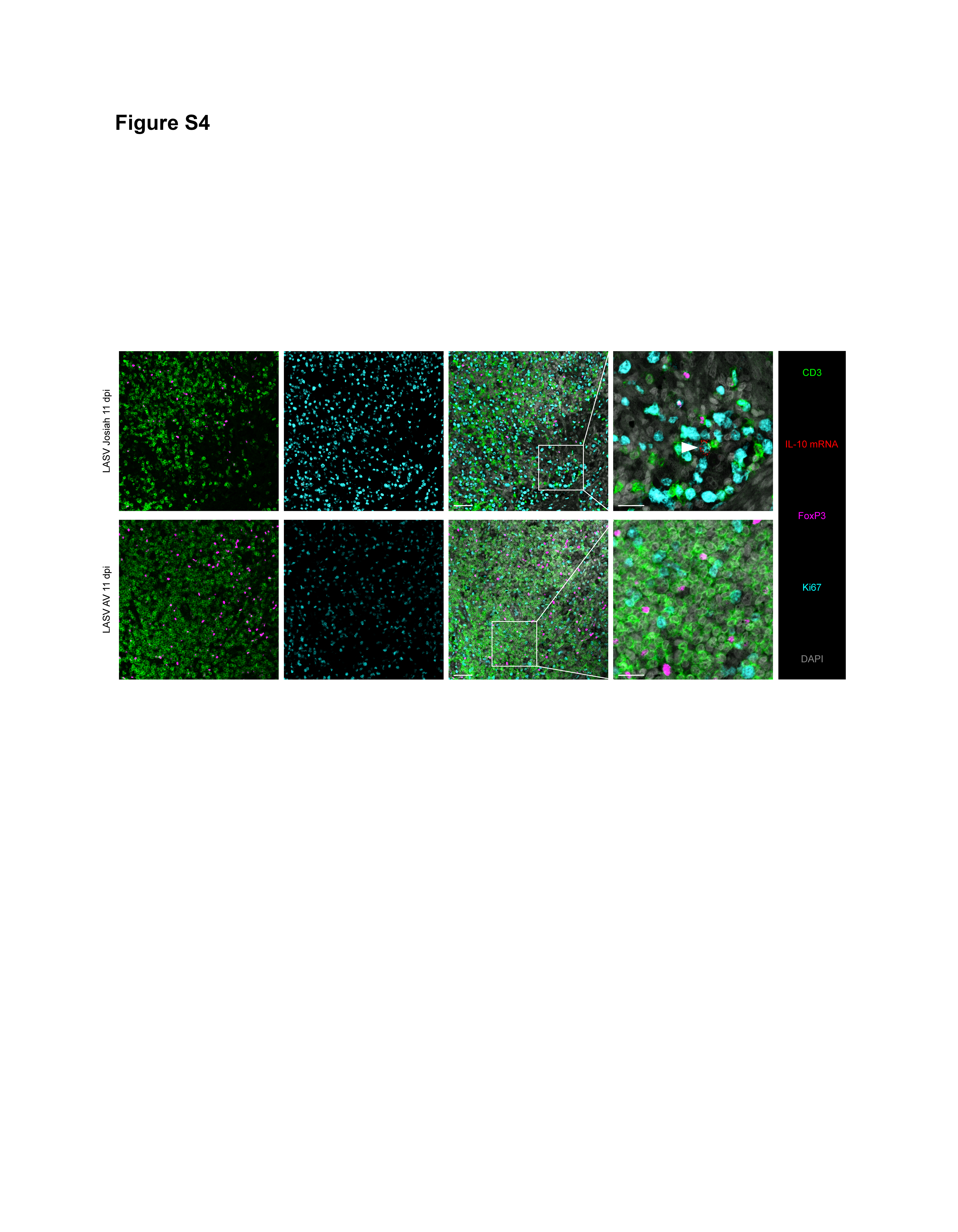

Supplement: S4 Fig — Confocal images illustrating the quantification presented in Fig 5F, allowing the visualization of Tregs (CD3+ FoxP3+, first column) and an unidentified cell positive for IL-10 mRNA (second column and white arrowhead in the fourth column). First column: merge of the markers CD3 and FoxP3, second column: merge of Ki67 and IL-10 mRNA, third column: merge of all four markers with DAPI staining, fourth column: higher magnification of the white squares. Scale of columns 1–3 = 50 µm and column 4 = 20 µm. (TIF) [file ppat.1013111.s004.tif]

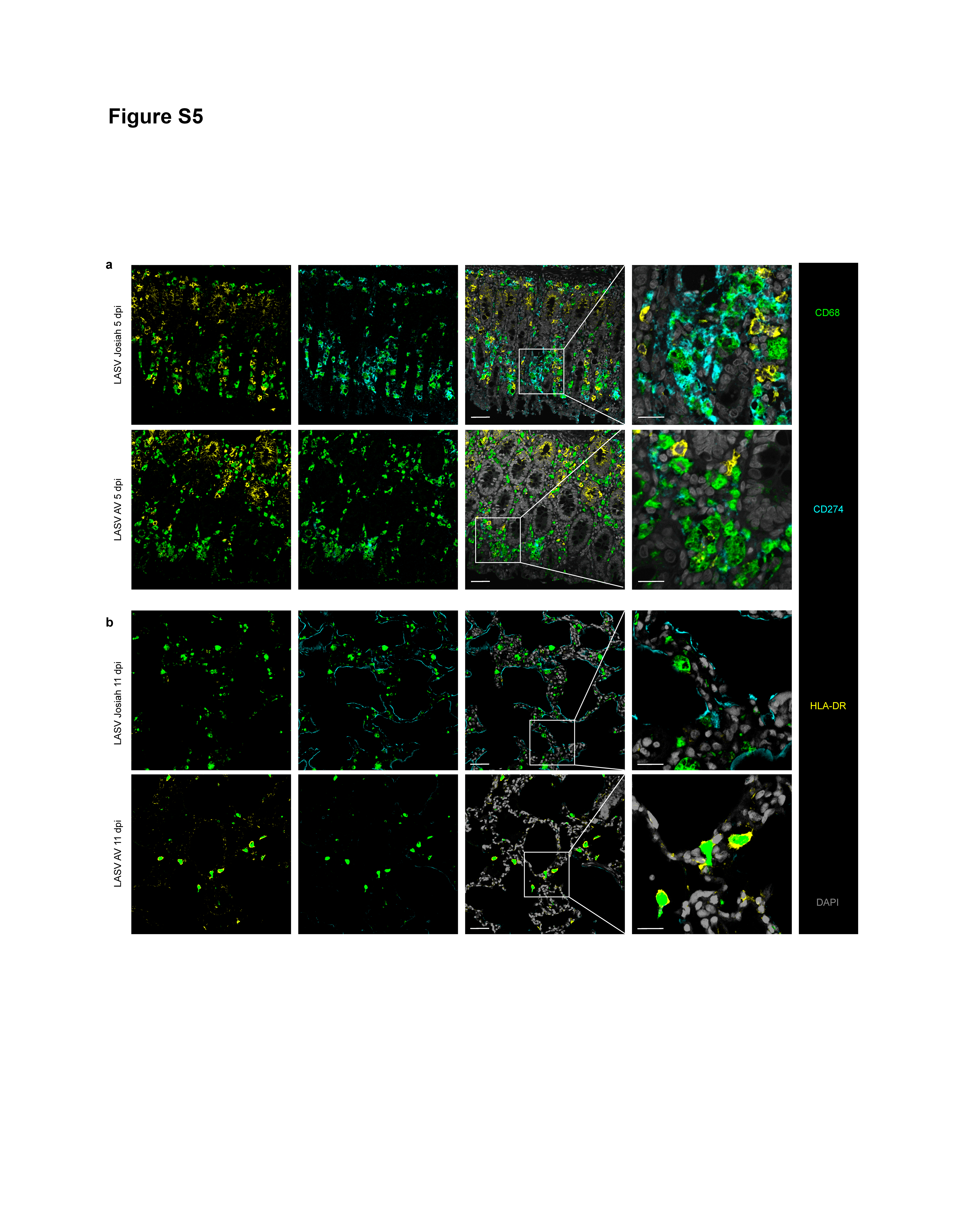

Supplement: S5 Fig — Confocal images illustrating the quantification presented in Fig 5G, allowing the visualization of CD68+ CD274+ HLA-DR- cells in the epithelial layer of the large intestine and the lung. First column: merge of the markers CD68 and HLA-DR, second column: merge of CD68 and CD274, third column: merge of all three markers with DAPI staining, fourth column: higher magnification of the white squares. Scale of column 1-3s = 50 µm and column 4 = 20 µm. (TIF) [file ppat.1013111.s005.tif]

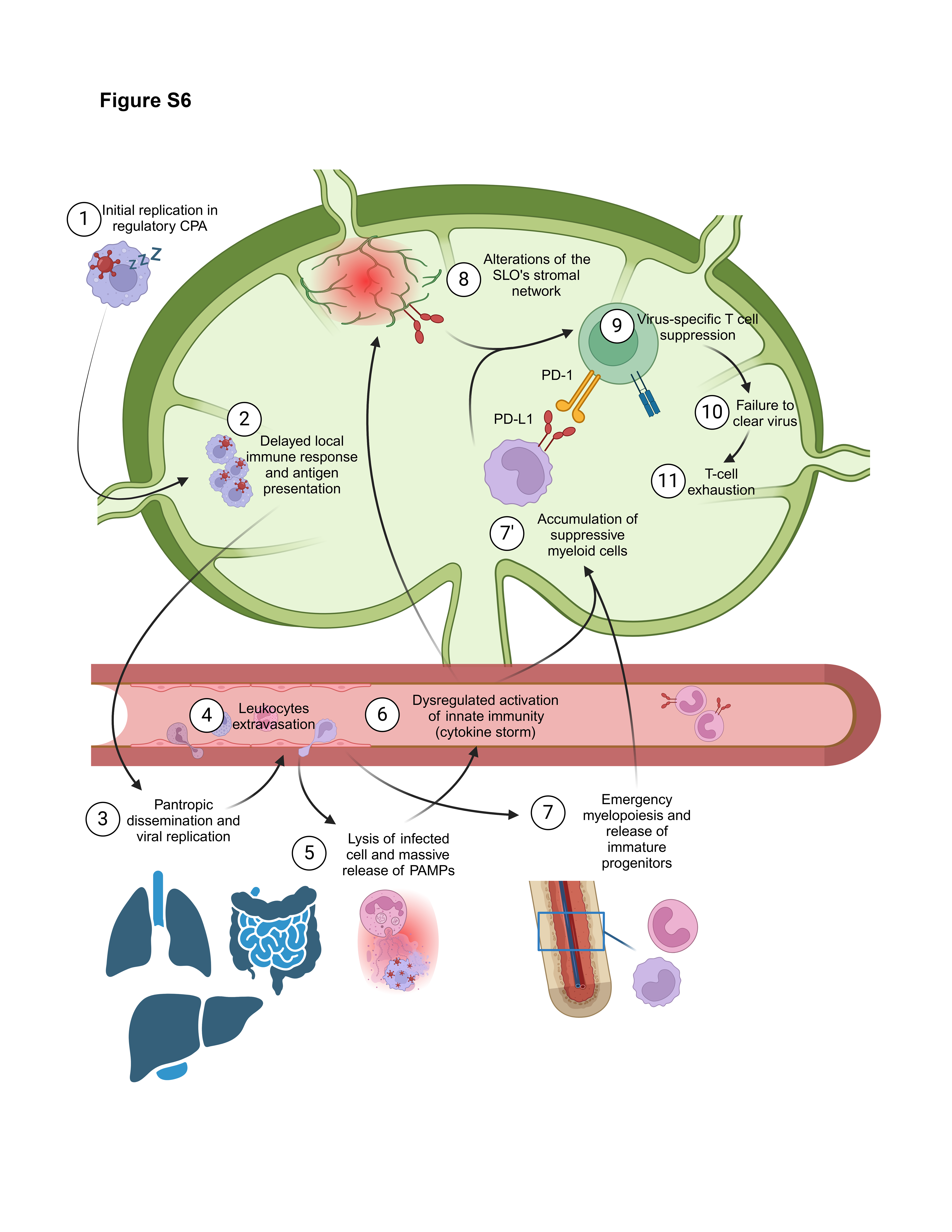

Supplement: S6 Fig — During fatal infection, the virus replicates rapidly in immunoregulatory macrophages, inducing defective priming of cellular adaptive immunity and allowing LASV to spread to peripheral organs and infect them. This pantropism induces the massive release of pathogen-associated molecular patterns (PAMPs), the recruitment of innate immune cells, and uncontrolled immune activation. Emergency myelopoiesis is initiated to replace the circulating pool of innate immune cells, leading to the release of immature myeloid cells that accumulate in the organs and further suppress the specific T-cell response. In parallel, alterations of the SLO stromal network impede T-cell homeostasis and activation, allowing relentless replication of the virus. Continuous contact with immunosuppressive myeloid cells and regulatory cytokines culminates in the exhaustion of primed T-cell clones and T-cell anergy. Created using BioRender under the CC BY 4.0 Licence. (TIF) [file ppat.1013111.s006.tif]
