# Supplementary material for: Expansion of myeloid suppressor cells and suppression of Lassa virus-specific T cells during fatal Lassa fever
Source: PLoS Pathog. 2025 Apr 17;21(4):e1013111. doi: 10.1371/journal.ppat.1013111 (PMC12040235; doi:10.1371/journal.ppat.1013111)
Supplement: S1 Table — (A) Antibodies and fluorophore panels used for histological studies. HIER buffer ER1 has a pH of 6 and ER2 has a pH of 9. Incubations with all primary antibodies were carried out for 30 min at RT, except CD279 for which the incubation was carried out for 1 h at RT. (B) Antibodies used for flow cytometry panels. Intracellular markers are in shown blue. (DOCX) [file ppat.1013111.s007.docx]

**Table S1**

1. **Antibodies and fluorophore panels used for histological studies**

| **Stromal network** | | | | | | |
| --- | --- | --- | --- | --- | --- | --- |
| Position | Antibody | Reference | Antibody dilution factor | HIER buffer | Opal fluorophore | Fluorophore dilution factor |
| 1 | Viral RNA ISH | RNAscope #463778 (Josiah) or #1156761 (AV) |  | ER2 | 650 | 750 |
| 2 | Granzyme B | Abcam #ab237847 | 100 | ER2 | 620 | 200 |
| 3 | CD274 | Abcam #ab228462 | 50 | ER2 | 570 | 110 |
| 4 | Desmin | Fisher Scientific #RB-9014-P1 | 500 | ER2 | 540 | 200 |
| 5 | cCasp3 | R&D Systems #MAB835 | 500 | ER1 | 520 | 250 |
| 6 | Calprotectin | Invitrogen #MA512213 | 500 | ER1 | 690 | 300 |
| **PD-1 PD-L1 interaction** | | | | | | |
| Position | Antibody | Reference | Antibody dilution factor | HIER buffer | Opal fluorophore | Fluorophore dilution factor |
| 1 | CD279 | Abcam #ab52587 | 50 | ER2 | 620 | 50 |
| 2 | CD274 | Abcam #ab228462 | 50 | ER2 | 570 | 120 |
| 3 | CD152 | Abcam #ab237712 | 250 | ER2 | 650 | 250 |
| 4 | CD3 | Dako #A045229-2 | 250 | ER2 | 540 | 200 |
| 5 | CD68 | Invitrogen #14-068882 | 500 | ER1 | 520 | 150 |
| 6 | HLA-DR | Abcam #ab92511 | 250 | ER2 | 690 | 200 |
| **Suppressive macrophages in peripheral organs** | | | | | | |
| Position | Antibody | Reference | Antibody dilution factor | HIER buffer | Opal fluorophore | Fluorophore dilution factor |
| 1 | CD274 | Abcam #ab228462 | 50 | ER2 | 650 | 150 |
| 2 | CD68 | Invitrogen #14-068882 | 500 | ER1 | 540 | 200 |
| 3 | HLA-DR | Abcam #ab92511 | 250 | ER2 | 520 | 400 |
| **Treg** | | | | | | |
| Position | Antibody | Reference | Antibody dilution factor | HIER buffer | Opal fluorophore | Fluorophore dilution factor |
| 1 | IL-10 ISH | RNAscope # 458118 | 50 | ER2 | 650 | 750 |
| 2 | CD3 | Dako #A045229-2 | 250 | ER2 | 540 | 200 |
| 3 | Ki67 | Abcam #ab15580 | 250 | ER1 | 690 | 200 |
| 4 | FoxP3 | Fisher Scientific #14-4777-82 | 250 | ER1 | 520 | 200 |

1. **Antibodies used for flow cytometry panels**

| Fluorophore | MDSC |  |  | Macrophages |  |  | T-cell activation |  |
| --- | --- | --- | --- | --- | --- | --- | --- | --- |
| BV421 | HLA-DR | Miltenyi 130-113-406 |  | HLA-DR | Miltenyi 130-113-406 |  | Ki67 | BD 562899 |
| BV510 | CD10 | BD 563032 |  |  |  |  | Live/Dead |  |
| FITC |  |  |  |  |  |  | CD137 | Miltenyi 130-119-886 |
| PE | CD279 | Biolegend 329906 |  |  |  |  | IFNg | BD 559327 |
| PE-CF594 | CD274 | Biolegend 329732 |  | CD274 | Biolegend 329732 |  |  |  |
| PE-Cy7 | CD66abce | Miltenyi 130-119-849 |  |  |  |  | CD154 | Miltenyi 130-113-608 |
| APC | CD33 | Miltenyi 130-113-345 |  |  |  |  | CD3 | BD 557597 |
| Alexa700 | CD11b | BD 557918 |  | CD163 | Fisher Scientific 56-1639-42 |  | CD4 | BD 560836 |
| APC-H7 | CD14 | Miltenyi 130-113-144 |  | CD14 | Miltenyi 130-113-144 |  | CD8 | BD 560179 |
