# Supplementary material for: Expansion of myeloid suppressor cells and suppression of Lassa virus-specific T cells during fatal Lassa fever
Source: PLoS Pathog. 2025 Apr 17;21(4):e1013111. doi: 10.1371/journal.ppat.1013111 (PMC12040235; doi:10.1371/journal.ppat.1013111)
Supplement: Attachment — Submitted filename: Responses to reviewers.pdf [file ppat.1013111.s011.pdf]

<u>Responses to reviewer’s comments</u>

We are very grateful to Dr Lukashevich and to the other reviewer for their helpful comments that substantially improved the manuscript. We have done our best to answer these comments.

Reviewers' Comments:

Part I – Summary

Reviewer #1: The aim of the current study is to provide evidence that recovery from acute LF is associated with LASV-specific CD4+ and CD8+ T-cell responses. The authors used frozen samples collected during 2021 Baillet N et al study to stimulate PBMC with a pool of overlapping peptides spanning the Josiah or AV GPC and NP in the presence of co-stimulatory antibodies and to count cytokine-secreting cells by flow cytometry. They found a gradual increase in the frequency of LASV-specific CD4+ and CD8+ T cells at 12 dpi and culminating at 32 dpi (Fig. 1A). By contrast, they did not observe any increase in the virus specific T-cells in Josiah-infected macaques. Using multiplexed immunofluorescence (IF) and in-situ hybridization (ISH) of histology sections of inguinal LN, the authors presented evidence that LASV Josiah infection was predominantly associated with DCs and immunoregulatory macrophages, while the AV strain was detected mostly in macrophages, endothelial, and stromal cells (Fig. 2). Transcriptomic analysis of the organs of infected animals and human PBMC-derived macrophages infected in vitro with Josiah and AV strains revealed more gene expression alterations during AV infection vs Josiah infection. Based on presented data, the authors hypothesized that bold inflammation observed in peripheral organs of Josiah-infected animals results in the depletion of mature monocytes and the expansion of immunosuppressive myeloid cells, alterations of LNs, and suppression of the virus-specific T-cell responses. In sum, presented data provides incremental contribution to better understanding of LF pathogenesis.

Reviewer #2: This article describes the immune response in a simian model of Lassa virus infection. This work complements two previously published articles by the same team on the same animals: Baillet et al., Comm Biol 2021, and Hortion J, Pos Pathog 2024. Here, they specifically describe the immunosuppressive aspect of this response, particularly in animals that do not survive in the lethal model (Josiah). The experiments and techniques used are appropriate, and the results obtained are thus clear-cut, allowing for a straightforward analysis of the findings. These results are clearly of great interest as they indicate the emergence of elements suggestive of immunosuppression in the group of non-surviving animals: a decrease in specific T cells, systemic dissemination of this immunosuppression (no compartmentalization), the appearance of MDSCs, immature neutrophils and HLA-DRlow monocytes, overexpression of PD-1 and PD-L1, and the disappearance of non-classical monocytes. As such, they deserve to be highlighted. That said, I have several remarks.

Part II – Major Issues: Key Experiments Required for Acceptance

Reviewer #1: Major concerns:

1. Study design. Sample collection procedures are poorly described. It looks like the most valuable samples for measuring LASV-specific T cell responses and histology (IF, ISH) analysis were collected during 2021 study (Baillet et al.). In this study, the latest point was 28 dpi. In the current study, the samples collected at 32 dpi were also analyzed. Are these late samples from the same or from “different” study? The authors must present clearly description of all samples used in this study with timing information and quality assessment.

We thank the reviewer for this comment. The samples are indeed the same as those described in the previous study (Baillet et al) and have been obtained at day 28 after infection, which was the day of necropsy of surviving animals. The '32' label on the x-axis of the PBMC graphs was an error and has been changed for 28. The x-axes of the graphs showing T-cell responses in SLOs were in contrast correctly labeled (28 DPI). All data presented in the current manuscript are new and were not published in the previous papers. We have added a sentence in the material and methods to clarify this point.

The authors used up to six cycles of immunodetection with same tissue sections by repeatedly stripping primary-secondary antibody complexes. Preservation of original antigen epitopes in heavily used tissue sections must be properly validated to exclude false-staining results. Why 6 cycles? Are staining patterns after two stripping procedures the same as after 6 cycles?

The protocols used on the BOND RXm follow the recommendations of the manufacturer (Leica) and of the provider of the staining reagents (Akoya). Successive strippings allow the horseradish peroxidase to be inactivated and the primary-secondary antibody complexes to be stripped, but also make certain epitopes that are difficult to label more accessible due to the fixation of organs in formalin. To perform a 6-plex labelling, it is necessary to remove the complexes after each labelling, resulting in 6 stripping operations. Setting up a 6-plex panel requires an antibody-by-antibody calibration to assess the number of stripping operations, the concentration and the type of stripping (Leica Epitope Retrieval Solution 1 or 2). Once each antibody has been calibrated, a combination of the different antibodies is tested and the quality of the labelling is compared with monolabelled slides to validate the panel or to continue optimizing the panel by changing the order of staining.

To illustrate the validation of the method, the figures below show the calcoprotectin staining (panel used in figure 5) either done after one stripping (left), 6 stripping steps with calprotectin staining performed during the 6th step and only calprotectin staining shown (center) or all staining shown (right) on serial sections. The morphology of the labelled cells and the fluorescence intensity are similar between the different conditions, confirming that the staining is not altered by the several stripping steps.

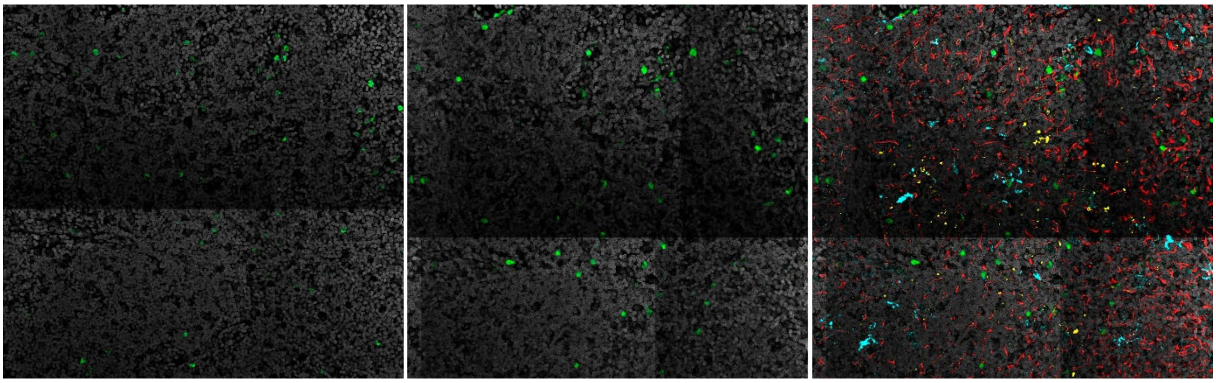

2. While the cell-mediated adaptive response plays a major role in the clearance of LASV during natural infection and/or vaccination, the data presented by the authors does not support this concept. The T cell kinetics data in Baillet et al (2021) publication and in current study (Fig. 1) are different and contradictory. In the Baillet et al (2021) study (Fig. 6), CD4+ and CD8+ T cells expressing functional markers of immune activation were picked at 12-15 dpi in AV-infected monkeys and declined at 28 dpi. This T cell response kinetics are in line with assumption that T cell responses play a major role in controlling viremia (picked on day 12). In the current study (using the same PBMC samples?), LASV AV-specific T cells were only barely detected on day 12 "and culminating at 32 dpi" (see below). As in case of neutralizing antibodies, these LASV-specific T cell responses seem to appear too late and in limited numbers to contain LASV replication. These contradictory T-cell results must be properly addressed and discussed.

The PBMC used for both studies are indeed the same ones, but the two data sets have to be compared cautiously. We have measured the expression of activation markers by whole circulating T cells, without peptide stimulation. The results are therefore not restricted to LASV-specific T cells. In this case, T-cell activation may for a main part result from bystander cells stimulated by non-specific signals, which is consistent with the large proportion of activated cells expressing KI67, perforin or Granzyme B. Moreover, the activation molecules measured in both studies are different and therefore may be expressed with a different kinetics. For example, CD69, which was detected as soon as 6 DPI in the previous study, is known to be one of the earliest activation markers transiently expressed by T cells and can be expressed when non-specific activation of T cells occurs. In addition, the kinetics of the activation markers measured in the previous study did not culminate at 12-15 DPI for all of them: CD69 peaked by day 6, KI67 by day 12-21 for CD8+ and day 21 for CD4+, perforin by day 9 for CD4+ and was increased from day 6 to 28 for CD8+. Nevertheless, we agree with the reviewer that these results are worth to be discussing. We therefore added a few sentences in the discussion section. Finally, we also agree that our data do not provide a demonstration that cell-mediated adaptive responses play a major role in the clearance of LASV in our model. The data only show an association between circulation of specific T cells and favorable outcome and do not rely on the presence of such T cells in tissues. We have further discussed this point in the discussion section.

Reviewer #2: (No Response)

Part III – Minor Issues: Editorial and Data Presentation Modifications
Please use this section for editorial suggestions as well as relatively minor modifications of existing data that would enhance clarity.

Reviewer #1: Minor concerns:

3. The NHP is the best experimental model to study LF pathogenesis. The route of the infection plays an important role in pathogenesis. Subcutaneous injections poorly mimic the natural LASV transmission. The authors must clearly articulate deficiency of their animal model. It is difficult to consider the inguinal LN as “the first site of infection” (see page 7, the last sentence).
Indeed, our model has limitations as we did not use the natural route of infection, which is the respiratory tract. We cannot infect NHP using aerosol route in our BSL4 facilities and this is the reason why we chose SC route that mimics a local infection rather than IM route that generates an immediate systemic dissemination of the virus. We therefore think that SC route is probably closer to the natural route of infection than IM. We have previously shown (Baillet 2021, Comm Biol) that LASV reaches the draining lymph node after the first cycles of replication that occur close to the injection site and that lymph nodes are probably a crucial site for viral amplification and may act as a viral reservoir allowing further systemic spreading. We have modified the sentence page 7 accordingly.

In fact, epithelial cells of the respiratory and/or digestive tracts are among the first host cells to interact with the virus during natural transmission/infection. Interaction with polarized epithelial cells is host-tissue and virus-strain specific and differently contributed to induction of innate immunity and LF pathogenesis. Experiments in NHPs provide evidence that interaction with mucosa of gastrointestinal tract and/or crossing epithelium barrier contributes to virus attenuation. Likewise, intracellular trafficking and interactions with innate immunity receptors are also strain-specific events that contribute to different patterns of innate immune responses and pathogenicity (e.g. Jonhson D et al., 2024). All these issues must be properly discussed.
We have added sentences to discuss this limitation of our model in the discussion section.

4. Page 7. At the middle of this page the authors noted, “a strong and gradual increase in the frequency of LASV-specific T cells …, starting at 12 dpi and culminating at 32 dpi (Fig. 1A)”. This notice is not correct. Since there are no samples/data available after 32 dpi, there is no experimental basis to support “culminating” claim. It is also incorrect regarding “gradual increase”, since LASV AV-specific CD8 T cells declined at 18 dpi in comparison with 12 dpi.
We agree that this sentence was incorrect and modified it accordingly.

5. The same page, last sentence. The authors stated that LN is the first site of infection where the immune response is primed. It suggests that LASV AV infection must be associated with favorable immune regulatory profile in the peripheral LN. However, as seen in Fig. 2d, immunosuppressive genes were strongly upregulated in AV infected LN vs samples collected from solid tissues.
We have removed the sentence about the priming of the immune response. Moreover, there was already a sentence in the discussion section about this observation: '*Surprisingly, these pathways appeared to be less strongly upregulated in the LNs of Josiah-infected animals than those of AV-infected animals at 11 DPI, perhaps because the virus-specific T-cell responses present in the AV-infected animals induce physiological regulatory mechanisms*'

6. It is also not clear why measurement of immunosuppressive “relevant genes in different organs” was limited to LN, intestine, and lung. Why did not measure in liver and spleen to keep at least partial consistency with data presented in panel D, Fig. 2?
We have measured the expression of the three genes in liver and spleen and added these results to figure 2D.

7. Page 12. The authors stated that “survival is associated in NHPs with the development of a LASV-specific T-cell response during the acute phase of the disease, whereas we did not detect such a response during lethal infection”. However, AV-specific T cell responses were only barely detected at 12 dpi and CD8+ T cells and declined at 18 dpi. There is no evidence that limited AV-specific CD8+ T cells really contribute to the viral control.
We have modified the text accordingly.

Reviewer #2: The most remarkable aspect of this study is the similarity of the results to those described in septic shock in human clinical cases. However, this aspect is only briefly mentioned, despite offering a major perspective for understanding the pathophysiology of severe infections, regardless of their origin. Here, the concept of viral sepsis should be considered. Numerous clinical references on bacterial sepsis should be added to the discussion to support this point, as this would reinforce the demonstration.
Indeed, we agree that severe VHF resembles to bacterial sepsis and the concept of viral sepsis clearly applies to Lassa fever. We have previously discussed this hypothesis using data obtained with the same model (Baillet 2021, Comm Biol). We have substantially improved the discussion section regarding this concept and added numerous references. There are now 14 references related to bacterial and viral sepsis in the manuscript.

In general, the manuscript should focus on the key findings that illustrate this similarity with septic shock. Too many results are presented, which dilute the main message. In this context, the results in Figure 3 do not contribute to the demonstration and should be removed.
We agree with the reviewer's comment and consequently moved Figure 3 to the supplementary information section. However, we think that these results deserve to be presented as they provide insights into the response of macrophages, which are key cells for immunosuppression, to LASV infection and into the role of viral strains in the phenotype induced. We have shortened the paragraph.

Do the authors have, among the numerous collected data, any results that could highlight an increase in regulatory T cells?

Data about regulatory T cells are already present in the manuscript, but there was no evidence of an increase of T reg during Lassa fever, whatever was the outcome. The percentage of T reg (CD3+ FoxP3+) in the lymph nodes of LASV-infected animals during the disease course is presented in Figure 3F (formerly 4F), and illustrations of the staining are presented in Figure S4 (formerly Fig. S3). Although we observed more T reg in Josiah-infected animals at day 2 whereas the opposite trend seems to occur by day 11, these differences were not significant. Moreover, the percentage of T reg remains very low in all conditions and quite close to the one measured in mock animals, suggesting that this T cell subpopulation does not play a major role in the immunosuppression. We added a few sentences in the result and discussion sections to further discuss this point.

The authors should be more cautious in their conclusions, as they do not establish a causal link between immunosuppression and the death of monkeys. The emergence of immunosuppression may simply be an indicator of an excessive inflammatory response (see septic shock mechanisms). In septic shock, immunosuppression is responsible for late mortality (i.e., after the first few days) by promoting nosocomial infections and prolonging ICU stays. However, here, no animal survives beyond day 12, making it difficult to extrapolate long-term impacts. While immunosuppression is likely partially detrimental, it is probable that mortality is primarily due to organ failure related to the inflammatory response, which in turn reflects the viral load progression in the early days (as described in Baillet et al.). The absence of medium-term survivors in the Josiah group is a limitation of the study that should be acknowledged.

We agree with the reviewer that the acute evolution of Lassa fever suggests that immunosuppression is probably not the main cause of fatal outcome and that reversing this phenomenon may not be sufficient to avoid such an evolution.

In connection with this last point, the timeline of mortality in Lassa fever clinical cases should be recalled to consider potential therapeutic options for survivors.

Done in the discussion section (last paragraph).

The authors' proposal to use anti-PD-1 may seem contradictory to certain recommendations advocating the use of anti-inflammatory treatments. How can these different approaches be reconciled?

Indeed, as for bacterial sepsis, potential therapeutics may be contradictory and their windows of use according to the disease course may be limited. For VHF, it seems important to limit the exacerbated and dysregulated inflammatory response but at the same time, it could be useful to try to restore specific T cell responses. It is the reason why a first evaluation of the different experimental therapies has to be done in a relevant animal model. We have added a sentence at the end of the discussion section to take this limitation into account.
