# Supplementary material for: Expansion of myeloid suppressor cells and suppression of Lassa virus-specific T cells during fatal Lassa fever
Source: PLoS Pathog. 2025 Apr 17;21(4):e1013111. doi: 10.1371/journal.ppat.1013111 (PMC12040235; doi:10.1371/journal.ppat.1013111)
Supplement: Attachment — Submitted filename: PPATHOGENS-D-25-00132.docx [file ppat.1013111.s009.docx]

Manuscript: PPATHOGENS-D-25-00132

Title: Expansion of myeloid suppressor cells and suppression of Lassa virus-specific T cells

during fatal Lassa fever

Authors: Blaise Lafoux et al.

This submission is a continuation of the efforts of Dr. Sylvain Baize group to elucidate pathogenesis of Lassa fever (LF) using non-human primates’ (NHPs) model in Cynomolgus monkeys. The authors used two different strains of Lassa virus (LASV), Josiah and AV, to induce fatal and non-fatal disease, respectively, after subcutaneous inoculation. In their previous publication in *Communications Biology* (Baillet N et al., 2021), the authors documented that high, uncontrolled viremia picked on day 12 and high AST in plasma correlated with clinical score and fatal outcomes in Josiah infected animals. Josiah infection resulted in high viral loads in numerous tissues. Cyto/chemokines in plasma and PBMC transcriptome profiles resembled bacterial septic-shock syndrome. In contrast, LASV AV infection induced modest viremia which declined after day 12. AST levels indicated limited functional damage in liver. The AV virus dissemination was detected predominantly in secondary lymphatic tissues. The authors suggested that “a robust CD4+ and CD8+ T cell responses, involving induction and proliferation of cytotoxic T cells” were responsible for effective viral load control and survival of AV-infected monkeys.

The aim of the current study is to provide evidence that recovery from acute LF is associated with LASV-specific CD4+ and CD8+ T-cell responses. The authors used frozen samples collected during 2021 Baillet N et al study to stimulate PBMC with a pool of overlapping peptides spanning the Josiah or AV GPC and NP in the presence of co-stimulatory antibodies and to count cytokine-secreting cells by flow cytometry. They found a gradual increase in the frequency of LASV-specific CD4+ and CD8+ T cells at 12 dpi and culminating at 32 dpi (Fig. 1A). By contrast, they did not observe any increase in the virus specific T-cells in Josiah-infected macaques. Using multiplexed immunofluorescence (IF) and in-situ hybridization (ISH) of histology sections of inguinal LN, the authors presented evidence that LASV Josiah infection was predominantly associated with DCs and immunoregulatory macrophages, while the AV strain was detected mostly in macrophages, endothelial, and stromal cells (Fig. 2). Transcriptomic analysis of the organs of infected animals and human PBMC-derived macrophages infected in vitro with Josiah and AV strains revealed more gene expression alterations during AV infection vs Josiah infection. Based on presented data, the authors hypothesized that bold inflammation observed in peripheral organs of Josiah-infected animals results in the depletion of mature monocytes and the expansion of immunosuppressive myeloid cells, alterations of LNs, and suppression of the virus-specific T-cell responses. In sum, presented data provides incremental contribution to better understanding of LF pathogenesis.

Major concerns:

1. Study design. Sample collection procedures are poorly described. It looks like the most valuable samples for measuring LASV-specific T cell responses and histology (IF, ISH) analysis were collected during 2021 study (Baillet et al.). In this study, the latest point was 28 dpi. In the current study, the samples collected at 32 dpi were also analyzed. Are these late samples from the same or from “different” study? The authors must present clearly description of all samples used in this study with timing information and quality assessment.

The authors used up to six cycles of immunodetection with same tissue sections by repeatedly stripping primary-secondary antibody complexes. Preservation of original antigen epitopes in heavily used tissue sections must be properly validated to exclude false-staining results. Why 6 cycles? Are staining patterns after two stripping procedures the same as after 6 cycles?

2. While the cell-mediated adaptive response plays a major role in the clearance of LASV during natural infection and/or vaccination, the data presented by the authors does not support this concept. The T cell kinetics data in Baillet et al (2021) publication and in current study (Fig. 1) are different and contradictory. In the Baillet et al (2021) study (Fig. 6), CD4+ and CD8+ T cells expressing functional markers of immune activation were picked at 12-15 dpi in AV-infected monkeys and declined at 28 dpi. These T cell response kinetics are in line with assumption that T cell responses play a major role in controlling viremia (picked on day 12). In the current study (using the same PBMC samples?), LASV AV-specific T cells were only barely detected on day 12 “and culminating at 32 dpi” (see below). As in case of neutralizing antibodies, these LASV-specific T cell responses seem to appear too late and in limited numbers to contain LASV replication. These contradictory T-cell results must be properly addressed and discussed.

Minor concerns:

3. The NHP is the best experimental model to study LF pathogenesis. The route of the infection plays an important role in pathogenesis. Subcutaneous injections poorly mimic the natural LASV transmission. The authors must clearly articulate deficiency of their animal model. It is difficult to consider the inguinal LN as “the first site of infection” (see page 7, the last sentence). In fact, epithelial cells of the respiratory and/or digestive tracts are among the first host cells to interact with the virus during natural transmission/infection. Interaction with polarized epithelial cells is host-tissue and virus-strain specific and differently contributed to induction of innate immunity and LF pathogenesis. Experiments in NHPs provide evidence that interaction with mucosa of gastrointestinal tract and/or crossing epithelium barrier contributes to virus attenuation. Likewise, intracellular trafficking and interactions with innate immunity receptors are also strain-specific events that contribute to different patterns of innate immune responses and pathogenicity (e.g. Jonhson D et al., 2024). All these issues must be properly discussed.
4. Page 7. At the middle of this page the authors noted, “a strong and gradual increase in the frequency of LASV-specific T cells …, starting at 12 dpi and culminating at 32 dpi (Fig. 1A)”. This notice is not correct. Since there are no samples/data available after 32 dpi, there is no experimental basis to support “culminating” claim. It is also incorrect regarding “gradual increase”, since LASV AV-specific CD8 T cells declined at 18 dpi in comparison with 12 dpi.
5. The same page, last sentence. The authors stated that LN is the first site of infection where the immune response is primed. It suggests that LASV AV infection must be associated with favorable immune regulatory profile in the peripheral LN. However, as seen in Fig. 2d, immunosuppressive genes were strongly upregulated in AV infected LN vs samples collected from solid tissues.
6. It is also not clear why measurement of immunosuppressive “relevant genes in different organs” was limited to LN, intestine, and lung. Why did not measure in liver and spleen to keep at least partial consistency with data presented in panel D, Fig. 2?
7. Page 12. The authors stated that “survival is associated in NHPs with the development of a LASV-specific T-cell response during the acute phase of the disease, whereas we did not detect such a response during lethal infection”. However, AV-specific T cell responses were only barely detected at 12 dpi and CD8+ T cells and declined at 18 dpi. There is no evidence that limited AV-specific CD8+ T cells really contribute to the viral control.
